# Transmission of unfolded protein response—a regulator of disease progression, severity, and spread in virus infections

**DOI:** 10.1128/mbio.03522-24

**Published:** 2025-01-08

**Authors:** Vibhu Prasad

**Affiliations:** 1 Department of Infectious Diseases, Molecular Virology, Center for Integrative Infectious Disease Research, Medical Faculty Heidelberg, Heidelberg University, Heidelberg, Germany; 2 Department of Microbiology and Molecular Medicine, Faculty of Medicine, University of Geneva, Geneva, Switzerland; Albert Einstein College of Medicine, Bronx, New York, USA

**Keywords:** unfolded protein response (UPR), virus infections, disease progression, cell-non-autonomous UPR, pro-viral state

## Abstract

The unfolded protein response (UPR) is a cell-autonomous stress response aimed at restoring homeostasis due to the accumulation of misfolded proteins in the endoplasmic reticulum (ER). Viruses often hijack the host cell machinery, leading to an accumulation of misfolded proteins in the ER. The cell-autonomous UPR is the immediate response of an infected cell to this stress, aiming to restore normal function by halting protein translation, degrading misfolded proteins, and activating signaling pathways that increase the production of molecular chaperones. The cell-non-autonomous UPR involves the spreading of UPR signals from initially stressed cells to neighboring unstressed cells that lack the stressor. Though viruses are known modulators of cell-autonomous UPR, recent advancements have highlighted that cell-non-autonomous UPR plays a critical role in elucidating how local infections cause systemic effects, thereby contributing to disease symptoms and progression. Additionally, by utilizing cell-non-autonomous UPR, viruses have devised novel strategies to establish a pro-viral state, promoting virus spread. This review discusses examples that have broadened the understanding of the role of UPR in virus infections and disease progression by looking beyond cell-autonomous to non-autonomous processes and mechanistic details of the inducers, spreaders, and receivers of UPR signals.

## UNFOLDED PROTEIN RESPONSE AND ITS ROLE IN HUMAN HEALTH AND DISEASE

**Fig 1:**
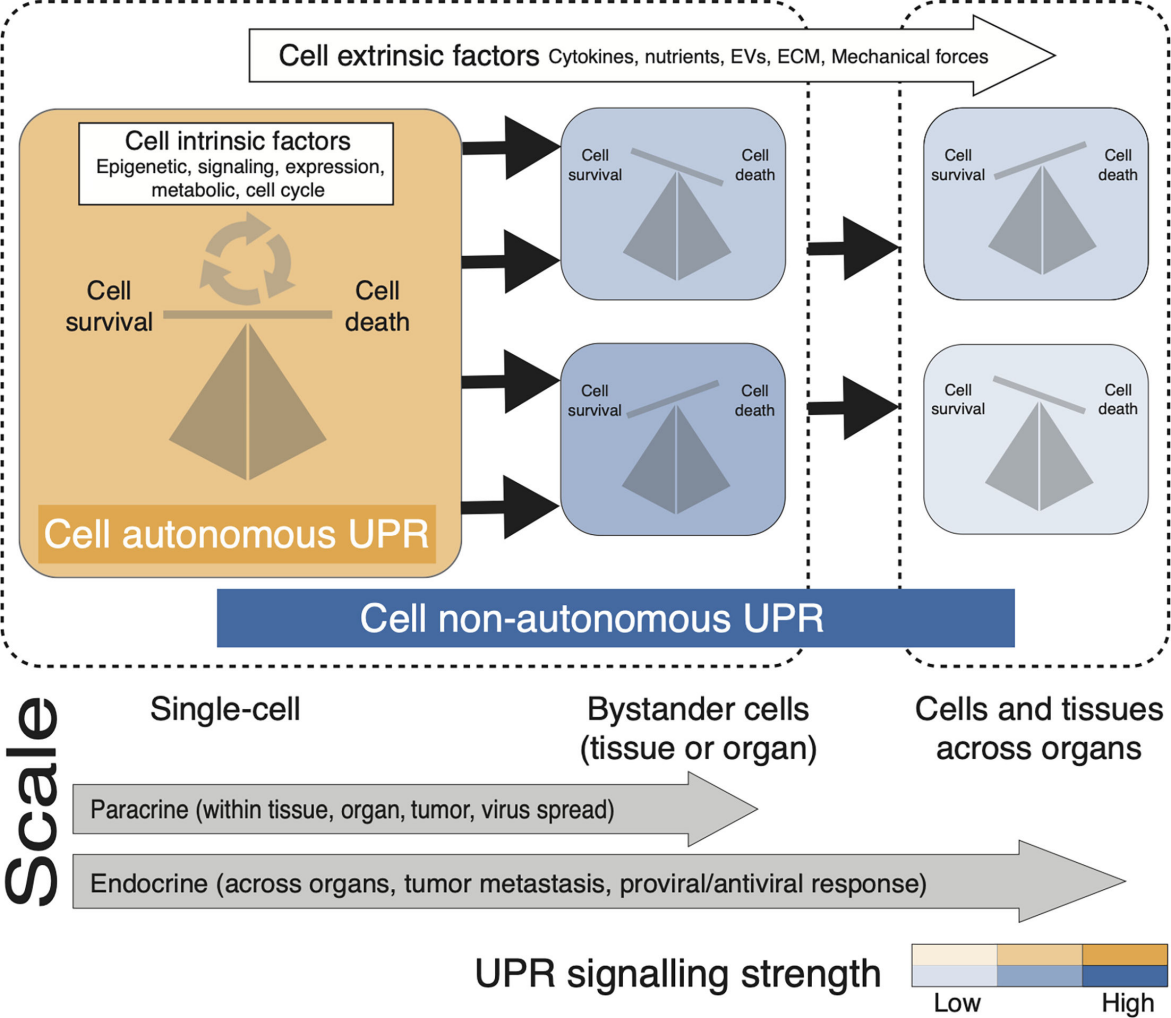
Cell-autonomous, non-autonomous UPR, and UPR signal heterogeneity—UPR signaling controls the fate of cell survival and death in primary UPR-initiating cells (yellow) and neighboring bystander cells (blue) via cell intrinsic and extrinsic factors and processes, respectively. Cell intrinsic factors are well characterized, but extrinsic factors are preliminary and speculative. The scale at the bottom of the figure indicates whether the cell-non-autonomous UPR acts in short distance (within tissue or organ) or long distance (across organs). Short-distance paracrine signaling in neighboring cells of the tissue occurs via secreted biomolecules. Long-distance endocrine signaling occurs across organs via bloodstream. The color scheme shows whether the UPR is induced in a cell via cell-autonomous (yellow) or non-autonomous (blue), whereas the intensity of the color shows whether UPR signaling is high or low. The differential UPR signaling in the producer and bystander cells of the same tissue or across organs in an organism signifies the heterogeneity of UPR signaling in a system.

The unfolded protein response (UPR) is a cellular stress response pathway activated by the accumulation of misfolded or unfolded proteins in the endoplasmic reticulum (ER) (1). The UPR aims to restore ER homeostasis by reducing the unfolded protein load and increasing the ER’s capacity to handle protein folding. It also initiates cell death pathways if the stress cannot be relieved, thereby regulating cell survival and death (Fig. 1). Detection of ER stress occurs at the ER membrane through transmembrane sensor proteins, categorized as protein kinase RNA-like ER kinase (PERK), inositol-requiring enzyme 1 (IRE1), and activating transcription factor 6 (ATF6). The UPR triggers transcriptional modifications by activating transcription factors such as activating transcription factor 4 (ATF4), X-box-binding protein 1 (XBP1), and the 50-kDa N-terminal fragment of ATF6 (ATF6-p50 or ATF6-N), facilitated by PERK, IRE1α (ubiquitously expressed in comparison to a paralog IRE1ß expressed in intestinal epithelial and mucosal cells [2]), and ATF6, respectively.

IRE1α is a highly conserved transmembrane protein found in both lower and higher eukaryotes. Upon ER stress, IRE1α undergoes phosphorylation and oligomerization, leading to conformational changes in its ribonuclease (RNase) domains (3). This activation allows IRE1α to cleave the unspliced mRNA of XBP1 (XBP1u), generating a transcriptionally active form known as XBP1s (spliced mRNA of XBP1). XBP1s plays a crucial role in regulating genes involved in ER stress relief, cell growth, differentiation, and survival. Two models of IRE1α activation have been proposed: one suggesting direct binding of unfolded proteins to IRE1α (3) and the other proposes indirect activation by relieving suppression mediated by ER-resident chaperone BiP (4). Downstream of IRE1, XBP1s transcriptionally upregulates UPR target genes and participates in the process of "regulated IRE1-dependent decay," contributing to ER quality control (5). Additionally, IRE1α can interact with tumor necrosis factor (TNF) receptor-associated factor 2 (TRAF2) to activate pro-apoptotic signaling pathways and facilitate ER-associated degradation (ERAD) of misfolded proteins (6). PERK, similar to IRE1, is a transmembrane protein with luminal stress sensing and cytoplasmic kinase domains. Upon ER stress, PERK undergoes oligomerization and autophosphorylation, leading to the phosphorylation of eukaryotic translation initiation factor 2A (eIF2α), which inhibits global protein translation but allows translation of select mRNAs (7). This selective translation allows for the preferential expression of, e.g., activating transcription factor 4 (ATF4), a transcription factor that regulates genes involved in apoptosis, including CAAT-enhancer binding protein homologous protein (CHOP) (8–10). PERK-mediated suppression of protein synthesis helps alleviate ER stress by reducing the influx of newly synthesized proteins into the ER, thereby preventing further accumulation of misfolded proteins and is termed as an integrated stress response (ISR) (11). The ISR coordinates various stress signals, including those from the ER, starvation, or heme depletion, converging on eIF2α phosphorylation and downstream signaling events (12). Finally, ATF6 serves as another class of ER stress sensor, undergoing proteolytic processing upon ER stress to generate an active transcription factor, ATF6-N (13). This processed form translocates to the nucleus and activates the expression of UPR target genes by binding to ER stress response elements in their promoters (14). ATF6-N regulates the expression of genes involved in ER stress response, including XBP1 and ER chaperone protein BiP (binding immunoglobulin protein), also known as GRP78 (glucose-regulated protein, 78 kDa) (15, 16), and can enhance CHOP levels to promote pro-apoptotic responses, albeit less efficiently than PERK (17). Further details of the mechanism of UPR sensor activation, functioning, and interactors are reviewed in References (18, 19).

A crucial regulator of the unfolded protein response (UPR) is the ER chaperone protein BiP/GRP78. BiP typically binds to the luminal domains of IRE1, ATF6, and PERK and prevents them from activation (20). When unfolded or misfolded proteins accumulate in the ER lumen, they displace BiP from these sensors, thereby activating them and initiating the UPR signaling cascade. The coordinated actions of IRE1, PERK, and ATF6 are vital for maintaining ER homeostasis, orchestrating adaptive responses to stress conditions, and modulating cellular fate in response to prolonged or severe stress (19). A functional UPR is essential for the well-being of multicellular organisms, as it regulates the balance between cell survival and cell death under stress conditions (Fig. 1) ([21). In a multicellular organism, which comprises different cell and tissue types forming various organs with specialized functions, the UPR operates through both cell-autonomous and non-autonomous modes. Cell-autonomous UPR involves individual cells activating UPR to alleviate ER stress by reducing protein synthesis, aiding protein folding, and enhancing the degradation of misfolded proteins. Conversely, growing evidence suggests the occurrence of cell-non-autonomous UPR, in which ER stress in one cell triggers UPR in neighboring or distant cells within the same organism, coordinating the cellular response across the organism (Fig. 1; discussed in section below). Next, I will discuss the intricacies of these UPR modes and how their dysregulation leads to detrimental diseases in humans.

### Cell-autonomous UPR maintains cellular homeostasis via cell intrinsic mechanisms

UPR induction in cells by various stimuli leads to the activation of sensor proteins and downstream activation of various signaling pathways, which are regulated via cell intrinsic mechanisms. To achieve homeostasis via cell intrinsic mechanisms, an activated UPR accelerates the folding of unfolded proteins by increasing the expression of chaperones (22), promoting the degradation of unfolded proteins by specialized processes like ERAD (23), and reducing the global protein synthesis to reduce the load of protein folding on ER (11). When the ER stress is intense, UPR can initiate autophagy to degrade and recycle excessive cellular components (1, 24). However, if the stress cannot be resolved in extreme cases, UPR can also initiate apoptosis to trigger programmed cell death to prevent damaged cells from affecting the tissue function (1, 24).

UPR signaling is a known modulator of innate immune pathways. UPR sensor IRE1α has been shown to activate nuclear factor-kappa B (NF-kB) signaling via interaction with TRAF2 protein on its cytoplasmic domain causing the removal of repressive IkB kinase from the cytoplasmic NF-kB subunits leading to its nuclear import and initiation of immune transcriptional response (25). Plasma cell differentiation-dependent activation of IRE1α–XBP1 was shown to be important for the levels of circulating antibodies (26). In dendritic cells (DCs), significant IRE1α basal activity causes constitutive XBP1s levels, which were shown to be important for the viability of DCs. XBP1s-deficient DCs showed increased apoptosis and decreased response to survival signals associated after Toll-like receptor (TLR) engagement (27). In addition, enhanced UPR signaling via IRE1α activation was shown to decrease major histocompatibility complex (MHC) class I presentation on the surface of DCs by promoting lipid accumulation, hence altering normal functioning of DCs (28, 29). The impact of UPR on cytokine production and tissue inflammation occurs via TLR engagement by agonists or lipopolysaccharides. which activates IRE1α–XBP1 (30). Mechanistically, direct recruitment of XBP1s to the enhancer region of interleukin-6 and TNF was shown to increase the expression and secretion of cytokines. The secreted cytokines can further modulate inflammation-induced UPR in target tissue by blocking or promoting UPR (31, 32).

Owing to the spectrum of physiological outcomes controlled by cell-autonomous UPR-activated pathways, it is unsurprising that dysregulation of cell-autonomous UPR has severe impact on human health via life-threatening diseases. These include metabolic diseases, e.g., diabetes and obesity, neurodegenerative diseases, e.g., Alzheimer’s, Parkinson’s, Huntington’s, and amyotrophic lateral sclerosis (ALS) involving the accumulation of misfolded proteins in neurons, neuronal cell death, and disease progression (24, 33, 34). For example, in ALS, the deregulation of proteostasis pathways leads to the accumulation of several host proteins including transactive response (TAR) DNA binding protein 43 or TDP43 in neurons (reviewed in reference 35). Other examples of diseases caused by defective cell-autonomous UPR are type II diabetes (36), Wolfram syndrome (37), and Wolcott–Rallison syndrome (38). Aberrant UPR was also shown to play a role in hepatocellular carcinoma, breast cancer, and colorectal cancer (34). Given the range of diseases affected by UPR, various UPR-based therapeutic strategies have been tested at the preclinical and clinical setup. In cancer, IRE1α RNase inhibitors STF-083010 and 4µ8C have shown efficacy in clinical cancer models (39, 40). Similarly, PERK inhibitors, e.g., GSK2606414, have been used in low amounts to be effective in mouse models of glucose-stimulated insulin secretion (41, 42), as well as attenuated pathological damage to hypothalamic neurons in a mouse model of tau protein-mediated dementia (43). There are also UPR inhibitors that are currently in clinical trials for some human diseases. For example, tauroursodeoxycholic acid (TUDCA), which is an endogenously produced bile acid from the conjugation of taurine with ursodeoxycholic acid (UDCA) in human liver, has been evaluated in Type 1 diabetes, hypertension, and cardiovascular diseases (34, 44), whereas UDCA is a clinically proven drug for the dissolution of cholesterol gallstones and hepatobiliary diseases (45). However, owing to the relatively high toxicity and limited efficacy *in vivo*, UPR-based therapeutic drugs have not progressed beyond preclinical studies (34). It is plausible that future identification of the messengers of cell-non-autonomous UPR with known therapeutic drugs will broaden the scope of inhibiting UPR and associated disease outcome.

### Cell-non-autonomous UPR or intercellular spread of UPR via cell-extrinsic mechanisms

One of the earliest evidence of cell-non-autonomous UPR transmission was described in a study by Mahadevan et al. (46) where the conditioned medium prepared from chemically induced UPR in tumor cells was shown to activate a generalized UPR in macrophages and pro-inflammatory cytokine signaling partly via Toll-like receptor 4 (TLR4) signaling. Although the messengers transmitting the UPR from tumor cells to macrophages were not defined, these data highlighted the potential role of cell-non-autonomous UPR to promote tumor growth, progression, and metastasis in a tumor microenvironment via macrophage-derived inflammation in a paracrine manner. The UPR-conditioned medium injected intraperitoneally to mice led to enhanced UPR in the liver suggesting that the mechanism can work via an endocrine system (Fig. 1). Recent studies have shown that, along with the neuronal cells in which accumulation of misfolded proteins cause disease, non-neuronal cells, like astrocytes and microglia, also contribute to disease progression via enhanced UPR through cell-non-autonomous mechanisms (47). In principle, non-autonomous UPR can be mediated through cell extrinsic mechanisms, including secreted cytokines, nutrients, extracellular vesicles (EVs), extracellular matrix (ECM) components, mechanical forces, and the intercellular signaling pathways that are modulated because of these factors (Fig. 1). These signaling molecules could induce UPR activation in neighboring cells to coordinate the cellular response to ER stress across a tissue or organism.

Cell-non-autonomous UPR or stress signaling can also operate when a tissue-specific UPR induction is recognized and acted upon at a systemic level to improve organism’s response to stress. This was shown in the worm *Caenorhabditis elegans* where endogenous expression of a temperature sensitive mutant protein expressed only in muscle tissue led to an increased expression of chaperone heat shock protein 90 (HSP90) to achieve proteostasis and enhanced folding in muscle tissue (48). Surprisingly, enhanced HSP90 expression was found not only in muscle tissue but also in intestinal or neuronal tissues indicating intercellular communication mediated cell-non-autonomous UPR aimed at improving global organismal proteostasis. A related study further showed that chaperones in Drosophila (Hsp40/70/90) can be secreted from cells via exosomes and can induce cell-non-autonomous UPR to promote protein folding environment in other tissues (49).

## MODULATION OF CELL-AUTONOMOUS UPR IN VIRUS INFECTIONS—A NEED FOR A BIGGER PICTURE?

Viruses infect cells and induce an environment conducive for their replication and amplification, but at the same time generate organismal stress as a direct response to infection. These stress responses activate immune responses that limits virus infections. Viruses counteract these responses by modulating the function or target of activated signaling pathways in order to benefit viral replication, evade immune responses, and restore homeostasis. Similar to other stress responses (e.g., oxidative or DNA damage response), homeostasis of the ER of an infected cell is achieved by cell-autonomous UPR via the concerted action of the sensor proteins and their downstream pathways. As viruses (especially enveloped viruses) require large amounts of viral membrane glycoproteins, this demand quickly overwhelms the host ER leading to global UPR via activation of all IRE1α, PERK, and ATF6 (Reviewed in (1, 50)). As continuous activation of UPR pathways can induce cell death, immune pathways, and global translation shutdown via PERK (1, 19), viruses have evolved strategies to modulate these pathways. These modulatory strategies by viruses involve both activation and suppression of UPR arms or downstream effectors.

Global activation of UPR by viruses, including dengue virus (DENV), African swine fever virus (ASFV), cytomegalovirus (CMV), hepatitis C virus (HCV), influenza A virus, Japanese encephalitis virus (JeV), vesicular stomatitis virus (VSV), leads to enhanced production of chaperones (calnexin, calreticulin, and BiP) (1, 50–53). In contrary, other viruses have been shown to activate a specific arm of UPR, for example, severe acute respiratory syndrome coronavirus (SARS-CoV) 8ab protein activates ATF6 (54), and adenovirus E3/19K glycoprotein activates IRE1α arm of UPR (55). Similar to the global or specific activation of UPR by the above viruses, suppression of UPR pathways is also common, confirming that balancing the ER stress response via activating and inhibitory strategies is the common theme of cell-autonomous UPR modulation by viruses. This defines an optimal environment for virus replication by perturbing, yet avoiding a blunting effect of cell innate immunity, autophagy, and apoptotic pathways. Similar to the evidences of UPR modulation by viruses as either a pro- or antiviral host response, UPR-activated immune response via antiviral or immune evasion mechanisms has also been shown in virus infections. As an antiviral mechanism, flavivirus-activated UPR signaling was shown to occur prior to the activation of innate immune signaling and synergized with a potent innate antiviral response (56). Chemical activation of UPR in cells before flavivirus infection led to an early innate immune signaling activation, suggesting that UPR cascades promoted antiviral immune response. In contrast, viruses also utilize UPR for evading immune response in host cells. For example, HCV activates UPR signaling specifically via IRE1α–XBP1s pathway, but XBP1s-dependent transactivation was blocked via unknown mechanisms (57). Some viruses also trigger the degradation of host immune components via ERAD pathways. For example, HCV-expressed proteins US2 and US11, as well as murine gammaherpes 68-expressed MK3 protein, interact and mislocalize MHC-I heavy chains for proteasomal degradation in the cytoplasm (58, 59). A similar degradation of host innate immune components (e.g., type I interferon receptor or IFNAR1) is induced by VSV- and HCV-infected cells via activation of the PERK signaling arm of UPR (1, 60).

Although UPR’s involvement in modulating the innate immune pathways in virus infections have been investigated, it is not clear whether cell-non-autonomous processes are involved in these perturbations and if specific immune responses are induced primarily in primary infected cells or bystander non-infected cells. This gap of information was highlighted, for example, during the recent global health emergency caused by coronavirus disease 2019 (COVID-19), where it became evident that virus titers in emerging infectious diseases do not always correlate with the outcome of the disease severity (61), suggesting that cells in which viruses are not replicating, known as bystander cells, also contribute to disease severity. The role of UPR in this was highlighted in a recent study where the monocytes from severe COVID-infected patients showed UPR component XBP1s among the top biological pathways involved in disease severity (62). Whether cell-autonomous UPR in primary infected lung tissue from COVID patients initiates a cell-non-autonomous UPR to impact the organismal response to virus infection remains to be seen. However, there is a growing need for broadening the scope of these investigations in virus infections.

## CELL-NON-AUTONOMOUS UPR IN VIRUS INFECTIONS: PROMOTING VIRUS SPREAD AND PATHOGENESIS

**Fig 2:**
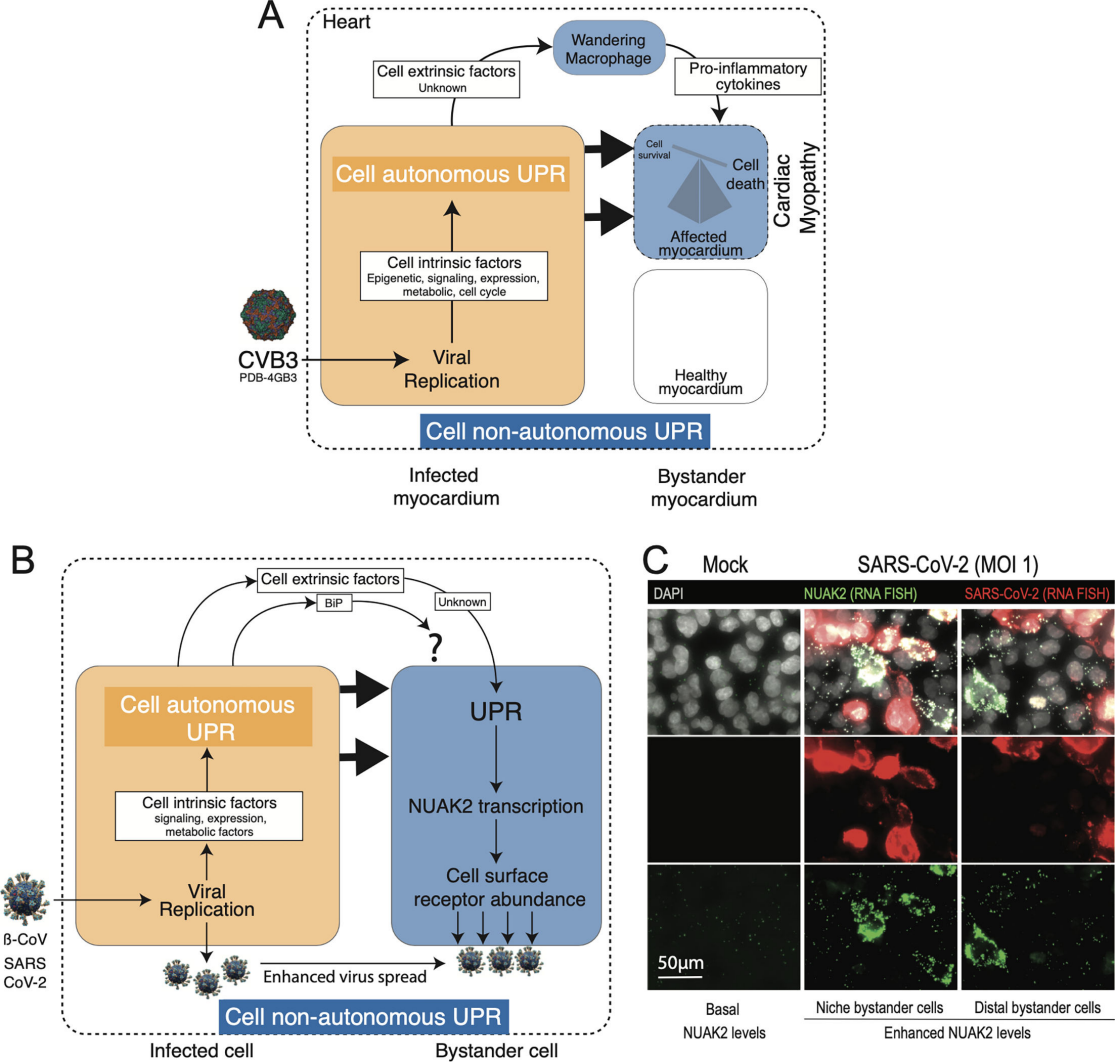
Cell-non-autonomous UPR in virus spread and pathogenicity—(**A**)Cell-non-autonomous UPR signaling promoting cardiac myopathy in CVB3-infected mice by transmission of UPR via unknown biomolecules from infected myocardium to wandering macrophages. The cell-autonomous UPR (yellow) activation in CVB3-infected myocardium promoted the release of unknown cell extrinsic factors that activated cell-non-autonomous UPR (blue) in wandering macrophages. This leads to pro-inflammatory cytokine secretion further activating the cell non-autonomous UPR in the neighboring healthy myocardium inducing cell death (cardiac myopathy) (65). (**B**)Cell-non-autonomous UPR in coronavirus (β-coronavirus and SARS-CoV-2) infection. SARS-CoV-2-infected lung epithelial cells show cell-autonomous UPR induction (yellow) in primary infected cells followed by transmission of cell-non-autonomous UPR (blue) to bystander cells via unknown biomolecules enhancing the pro-viral kinase NUAK2 that increases receptor abundance promoting the binding and uptake of SARS-CoV-2 (66). ß-coronaviruses promote the secretion of BiP from infected cells (67). (C)Niche and distal bystander non-infected cells with enhanced NUAK2 expression separated from SARS-CoV-2-infected cells using single-cell RNA-fluorescence *in situ* hybridization (FISH). Data are related to the study of Prasad et al. (66).

Recently, a direct example of cell-non-autonomous UPR and its impact on virus pathogenesis was shown during the coxsackievirus B3 (CVB3)-infection of heart muscles (myocardium), a condition that contributes to viral myocarditis, which is an inflammatory condition of the myocardium promoted by virus-induced ER stress (63). Lytic and persistent CVB3 infection may promote cardiomyocyte damage leading to acute or persistent myocarditis, respectively (64). The severity of this effect was attributed to the transfer of ER stress from initially infected heart muscles to the wandering macrophages, which led to enhanced pro-inflammatory cytokine production and worsening of viral myocarditis (65). The study highlighted that ER stress induction in macrophages was not directly initiated by viral infection but in a cell-non-autonomous manner via unknown biomolecules secreted from infected and ER-stressed myocardiocytes (Fig. 2A). Further analysis showed that CVB3-infected myocardiocytes could transmit the ER stress to macrophages through trans-well inserts suggesting that soluble factors within the cardiac microenvironment are sufficient to transfer the ER stress. Though previous studies highlighted the importance of TLR4 signaling cascade in activating the selective cell-non-autonomous UPR in macrophages (30, 46), the transmission of ER stress from CVB3-infected myocardiocytes to macrophages was not affected by inhibition of TLR4 signaling in macrophages. Overall, these findings highlighted that several unknown biomolecules from producer cells can transmit UPR to bystander cells via receptors and signaling cascades that depend on the intensity (acute or continuous) and nature (chemical or physiological) of UPR inducer in producer cells.

Another direct evidence for virus-induced cell-non-autonomous UPR promoting viral spread was recently shown by us for SARS-CoV-2 (66). In a differential transcriptomic-based analysis, we identified the cellular factors that were enhanced both in the conventional and SARS-CoV-2-induced UPR. While screening for their role in SARS-CoV-2 infection, we identified that NUAK2 (novel [nua] kinase 2), an AMPK (adenosine monophosphate-activated protein kinase)-related kinase, played an important role in promoting the binding and uptake of SARS-CoV-2 virions to cells by regulating the expression of global cell surface receptors, including the SARS-CoV-2 receptor angiotensin-converting enzyme 2 (ACE2). However, since the activation of NUAK2 in initially infected cells occurred after viral replication, its role in promoting viral entry appeared contradictory. Further results clarified that NUAK2 increase occurred both in initially infected as well as bystander non-infected cells, thereby promoting receptor abundance and virus uptake in bystander cells and enhancing viral spread. The study further showed that the underlying reason for NUAK2 increase in bystander cells was the transmission of UPR from initially infected cells to bystander cells via unknown biomolecules (Fig. 2B). These results indicated that cell-non-autonomous UPR can be utilized by viruses to induce a pro-viral environment in a tissue resulting in enhanced viral spread. The biomolecules involved in the transmission of UPR to bystander cells is an important aspect for future investigation. The data from the study suggested that the bystander cells with activated UPR or enhanced NUAK2 levels appeared to be located either in niche or distant cells relative to the initially infected cells (Fig. 2C). Future studies would need to address (i) the nature of the biomolecular entity (RNA, protein, or lipid) involved in this transmission and (ii) whether the transmission of biomolecules occurred through cell-to-cell contact (niche) ECM-associated soluble molecules or via extracellular vesicle (EV)-associated material or both. This will clarify whether the non-autonomous UPR in SARS-CoV-2 infection plays a role in local lung tissue inflammation or long-range inflammatory responses reported for SARS-CoV-2 (67).

In addition to the direct examples discussed above, a few studies have indirectly reported on the role of virus-induced cell-autonomous UPR in infected cells and its potential in modulating bystander cell-non-autonomous UPR via secreted biomolecules promoting viral pathogenicity or infection spread. The potential induction of cell-non-autonomous UPR in β-coronavirus-infected cells was indirectly shown in a recent study where the egress of virus from infected cells was shown to occur concomitantly with components of UPR signaling, e.g., ER-localized chaperone BiP/GRP78 (68). Though the relevance of co-secretion of BiP with β-coronavirus virions was not clarified (Fig. 2B), there might be two reasons why this can be a potential modulator of cell-non-autonomous UPR during infection as follows: (i) As secreted chaperones are known inducers of cell-non-autonomous UPR (49), BiP secreted during β-coronavirus infection might be a potential inducer of cell-non-autonomous UPR, perturbing organismal homeostasis and inducing systemic immune response to promote disease severity. (ii) Since BiP was shown to modulate virus-induced pathogenicity by interacting and controlling the secretion of a viral pathogenicity factor, e.g., NS1 secretion in Dengue infection (69), it is plausible that secretion of BiP during infection may promote the co-secretion of other cellular and viral proteins important for viral spread or pathogenesis via cell-non-autonomous UPR induction. Yet, the mechanism of escape of BiP from the ER lumen and its secretion is unclear. One plausibility could be that the cleavage of BiP and subsequent removal of its C-terminal ER retention signal, as shown for AB5 toxins from Shiga, cholera, pertussis, and subtilase toxins produced by toxigenic strains of *Escherichia coli* bacterium (70), could trigger its secretion leading to the activation of all UPR sensors (71). In this regard, investigating whether secreted BiP is present in the cleaved form under different UPR inductions may shed light on its secretion mechanism and role in cell-non-autonomous UPR.

One way the cell-autonomous UPR could promote non-autonomous UPR in neighboring cells or tissue is via secreted biomolecules (Fig. 1). Hence, viruses modulating the secretome of infected cells can speculatively induce cell-non-autonomous UPR. In this regard, various studies have highlighted the role of virus infection-induced secretome in modulating the microenvironment of infected tissue for anti-viral, pro-viral, oncolytic, and immune activation outcomes by either investigating how virus replication is affected by globally secreted human proteins or by measuring the secretome of virus-infected cells. For example, a targeted screen looked at the role of known human secreted proteins in vesicular stomatitis virus (VSV) replication and identified fibroblast growth factor 16 (FGF16) as an inhibitor of VSV replication (72). Another study measured the secretome of Porcine reproductive and respiratory syndrome virus-infected cells using stable isotope labeling with amino acid in cell culture-based proteomic analyses and found that most of the secreted proteins from infected cells use nonclassical secretory pathways (73). However, the role of these secreted proteins in the virus infection was not defined. A recent study showed that the secretome of human cytomegalovirus (HCMV)-infected cells had cellular factors that promoted angiogenesis and wound healing, thereby indirectly promoting a vascular disease (74). In a similar report, transmitted factors from primary infected HCMV modulated the bystander cells to promote virus infection and spread (75). Herpes Simplex Virus-1 (HSV-1)-infected cells showed enhanced secretion of host proteins including filamin, tubulin, heat shock proteins (HSPs), ECM proteins were downregulated (76). Similarly, influenza infection-induced secretome was shown to promote bystander cell pathogenesis in cell culture studies via leakage of rapid lysosomal proteins. This subsequently led to inflammasome activation and apoptosis (77). Given the role of induced UPR in bystander cells promoting virus spread or cell death (65, 66), UPR might be involved in the bystander cell effects seen in these cases of virus-infected cells.

Overall, only a few direct and some indirect findings reported above have unveiled how cell-non-autonomous UPR during virus infections is critical for understanding the full extent of virus infection-induced pathogenicity and disease severity, especially for emerging infectious agents. Future work will address mechanisms and messengers of cell-non-autonomous UPR in virus infection spread and pathogenesis.

## OUTLOOK: HETEROGENEITY IN UPR SIGNALING VIA CELL-AUTONOMOUS AND NON-AUTONOMOUS UPR MECHANISMS AND ITS IMPORTANCE FOR VIRUSES

Recent results showing the relevance of cell-non-autonomous UPR in promoting disease severity and spread of virus-infected cells have brought to focus new research avenues of combining cell-autonomous with -non-autonomous UPR to find the full extent of UPR signaling in virus infection. First, it is important to distinguish virus-induced cell-non-autonomous effects to be UPR-dependent or -independent. Second, the use of physiological cues for UPR induction, for example, ER overload by protein polymers and inclusion forms like Hong Kong null mutant of alpha antitrypsin (78), or stress response during hepatic regeneration (79), will help mimic how UPR works in systemic states through cell-autonomous and -non-autonomous processes. Third, the heterogeneity of UPR in virus infection and its relevance to the infection outcome should be investigated (80). It is known that the interplay of cell-autonomous and -non-autonomous processes can lead to heterogeneity in cellular response to signaling by influencing the microenvironment surrounding the cells and thereby modulating the signaling pathways within these cells (81). Similarly, in the case of UPR, biomolecules transmitting the UPR response to bystander cells via paracrine signaling could lead to heterogenous responses. For example, UPR originating in virus-infected cells could lead to the secretion of variable levels of biomolecules that will likely induce a stronger response in cells that are in proximity to the infected cells that are more distant. Also, cell-to-cell interactions and extracellular cues depending on the microenvironment of cells can result in a heterogenous UPR. Hence, heterogeneity in UPR signaling and UPR spread might also contribute to variability in virus infection and spread.

For viral infection at the local tissue level, a recently reported method that can be utilized for interplay of cell-autonomous and -non-autonomous UPR is spatial transcriptomics. In this method, cell types can be assigned to their location in a histological tissue section together with mRNA information at genome-wide scale. Using this methodology, identification of novel phenomena, like viral permissiveness, and cellular factors, like NUAK2 that promote pro-viral signaling in cells via UPR can be achieved in an efficient and broader scale. While cell-to-cell communication plays a pivotal role in coordinating immune responses during viral infections via anti-viral signaling, limited attention has been given to the pro-viral condition that supports viral replication. The perturbation of cell-autonomous UPR by viruses could govern the selected secretion of cytokines and biomolecules that may direct an anti or pro-viral signaling state in bystander cells. In this regard, already available data from virus secretome studies can be overlapped with UPR secretome to identify unconventional messengers and modulators of UPR signaling. This will also help in separating the UPR-dependent and -independent biomolecules and messengers in affecting the virus spread and disease severity.

## CONCLUSIONS

Cell-to-cell communication, as well as its impact on disease progression, modulation of microenvironment by tumors, and virus infection dynamics, is still in its infancy. Viruses modulate direct ER stress response elicited in an infected cell (cell-autonomous UPR), but recent evidence has shown that viruses also modulate the transmission of message from initially stressed cells to neighboring bystander cells (cell-non-autonomous UPR) to their advantage. Cell-non-autonomous UPR induced by secreted cellular factors plays an important role in progression of human diseases, cancer, and as evidenced by recent data, also in virus spread and viral disease severity. Viral examples have highlighted the importance of cell-non-autonomous UPR in enhancing the severity of virus disease in CVB3-induced myocardiopathy (65) and SARS-CoV-2-induced pro-viral state for enhanced virus spread via NUAK2 kinase (66). These studies underscore the importance of future efforts to better understand the contribution of cell-non-autonomous UPR in virus–host interplay.
